# Cortical thickness in chronic pain: A protocol for systematic review and meta-analysis

**DOI:** 10.1097/MD.0000000000021499

**Published:** 2020-07-31

**Authors:** HaiRong Ma, LiQin Sheng, Fei Chen, CongHu Yuan, ZhenYu Dai, PingLei Pan

**Affiliations:** a Department of Neurology, Kunshan Hospital of Traditional Chinese Medicine, Jiangsu; b Department of Radiology; c Department of Anesthesia and Pain Management; d Department of Neurology; e Department of Central Laboratory, Affiliated Yancheng Hospital, School of Medicine, Southeast University, Yancheng, PR China.

**Keywords:** chronic pain, coordinate-based meta-analysis, cortical thickness, gray matter, seed-based d mapping with permutation of subject images

## Abstract

**Background:**

Numerous studies using a variety of non-invasive neuroimaging techniques in vivo have demonstrated that chronic pain (CP) is associated with brain alterations. Cortical thickness (CTh) via surface-based morphometry (SBM) analysis of magnetic resonance imaging data is a valid and sensitive method to investigate the structure of brain gray matter. Many studies have employed SBM to measure CTh difference between patients with CP and pain-free controls and provided important insights into the brain basis of CP. However, the findings from these studies were inconsistent and have not been quantitatively reviewed.

**Methods:**

Three major electronic medical databases: PubMed, Web of Science, and Embase were searched for eligible studies published in English on April 3, 2020. This protocol was prepared based on the Preferred Reporting Items for Systematic review and Meta-Analysis Protocols. The Seed-based *d* Mapping with Permutation of Subject Images software package will be employed to conducted a coordinate-based meta-analysis (CBMA) to identify consistent CTh differences between patients with CP and pain-free controls. Several complementary analyses, including sensitivity analysis, heterogeneity analysis, publication bias, subgroup analysis, and meta-regression analysis, will be further conducted to test the robustness of the results.

**Results:**

This CBMA will tell us whether CP with different subtypes shares common CTh alterations and what the pattern of its characterized alterations is.

**Conclusions:**

To the best of our knowledge, this will be the first CBMA of SBM studies that characterizes brain CTh alterations in CP. The CBMA will provide the quantitative evidence of common brain cortical morphometry of CP. The findings will help us to understand the neural basis underlying CP.

**Trial Registration number:**

INPLASY202050069

## 1 Introduction

Chronic pain (CP) is defined as pain that persists or recurs for more than 3 months.^[1,2]^ CP is highly prevalent worldwide and has emerged as a major global public health.^[3,4]^ CP adversely affects a person's physical function and quality of life and causes a substantial societal economic burden.^[3,4]^ Although CP is heterogeneous in forms and in etiologies; there is convergent evidence that CP may share a common pathophysiology associated with central nervous system reorganizations. Numerous studies using a variety of non-invasive neuroimaging techniques in vivo have demonstrated CP-related brain alterations in the neurochemical profile, regional gray matter (GM), regional spontaneous activity, functional connectivity and networks.^[5–16]^ The key altered brain areas, including the sensorimotor, prefrontal, cingulate, and insular cortices in the sensorimotor network, default mode network, and salience network, were not only involved in sensory processing, but also in cognitive-affective processing, which have helped us to understand the maladaptive neurobiological mechanisms leading to the development of CP.^[17]^

Cortical thickness (CTh) via surface-based morphometry (SBM) analysis of high-resolution 3-dimensional anatomical magnetic resonance imaging (MRI) data is a valid method to study the structure of brain GM.^[18]^ Compared to voxel-based morphometry (VBM) that provides a mixed measure of GM including cortical surface area or cortical folding as well as cortical thickness,^[18]^ SBM may be more sensitive to detect subtle brain structural differences between groups.^[18,19]^ VBM and SBM are complementary methods for the observation of brain morphometry.^[18,20,21]^ Several meta-analyses of VBM studies have shown consistent evidence of GM volume/density alterations in CP.^[5–10,22,23]^ In the last decade, increasing studies have employed SBM to measure CTh difference between patients with CP and pain-free controls and provided important insights into the brain basis of CP. However, the findings from these studies were inconsistent and have not been quantitatively reviewed. It remains unknown whether CP with different subtypes shares common CTh alterations and what the pattern of its characterized alterations is. For this purpose, meta-analysis is essential for the synthesis of the findings from these CTh studies.

Coordinate-based meta-analysis (CBMA) is a useful technique to detect consistency of brain alterations across neuroimaging studies in a particular disorder for a specific question. In the present study, we will use Seed-based d Mapping with Permutation of Subject Images (SDM-PSI)^[24,25]^ to perform this CBMA of CTh studies in CP.

## 2 Methods

### 2.1 Literature search strategies

Three major electronic medical databases: PubMed, Web of Science, and Embase were searched for eligible studies published in English from each database's inception to April 3, 2020. The following terms were used for the searches: ((chronic pain) OR (chronic myofascial pain) OR (chronic headache∗) OR (chronic migraine∗) OR (burning mouth syndrome) OR (temporomandibular joint disorder∗) OR (neck pain) OR (shoulder pain) OR (phantom limb pain) OR (chronic thoracic pain) OR (chronic chest pain) OR (chronic back pain) OR (chronic knee pain) OR (chronic ankle pain) OR (chronic epicondylalgia∗) OR (chronic abdominal pain) OR (chronic visceral pain) OR (chronic pelvic pain syndrome) OR (neuropathic pain) OR (trigeminal neuralgia) OR neuralgia OR (postherpetic neuralgia) OR (complex regional pain syndrome) OR fibromyalgia OR (ankylosing spondylitis) OR (chronic epigastric pain syndrome) OR (irritable bowel syndrome) OR (inflammatory bowel disease) OR (Crohn disease) OR (chronic bladder pain syndrome) OR (chronic testicular pain) OR (functional dyspepsia) OR (musculoskeletal pain) OR (chronic widespread pain) OR (chronic whiplash-associated disorder) OR arthritis OR (somatoform pain) AND ((cortical thickness) OR (cortical thinning) OR (surface-based morphometry)). Neither article language nor publication time was limited. In addition, manual searches were conducted within the reference lists of the included studies and any relevant review articles.

This protocol was prepared based on the Preferred Reporting Items for Systematic review and Meta-Analysis Protocols (PRISMA-P).^[26]^

### 2.2 Eligibility criteria

#### 2.2.1 Inclusion criteria

The studies have to meet the following inclusion criteria:

(1) studies that investigated regional CTh differences between patients with CP and matched pain-free controls at the whole-brain cortical level;
(2) studies with non-significant results and studies with significant findings that reported brain clusters in standard Montreal Neurological Institute (MNI) or Talairach space;
(3) an original article published in English in a peer-reviewed journal.

#### 2.2.2 Exclusion criteria

Publications will be excluded if:

(1) the sample size was fewer than 7 either in the CP group or the pain-free group;
(2) three-dimensional coordinates of significant CTh results were not reported;
(3) the studies only employed regions of interest analysis or global CTh analysis;
(4) a direct pain-free compassion group was lacked;
(5) the patient sample was overlapped with the another one with a larger sample size;
(6) no baseline comparison was performed in case of a longitudinal study;
(7) the pain duration was less than 3 months;
(8) studies investigated experimental pain or acute pain;
(9) the publications were conference abstracts, research protocols, case reports, letters, reviews, and editorials.

**Figure 1:**
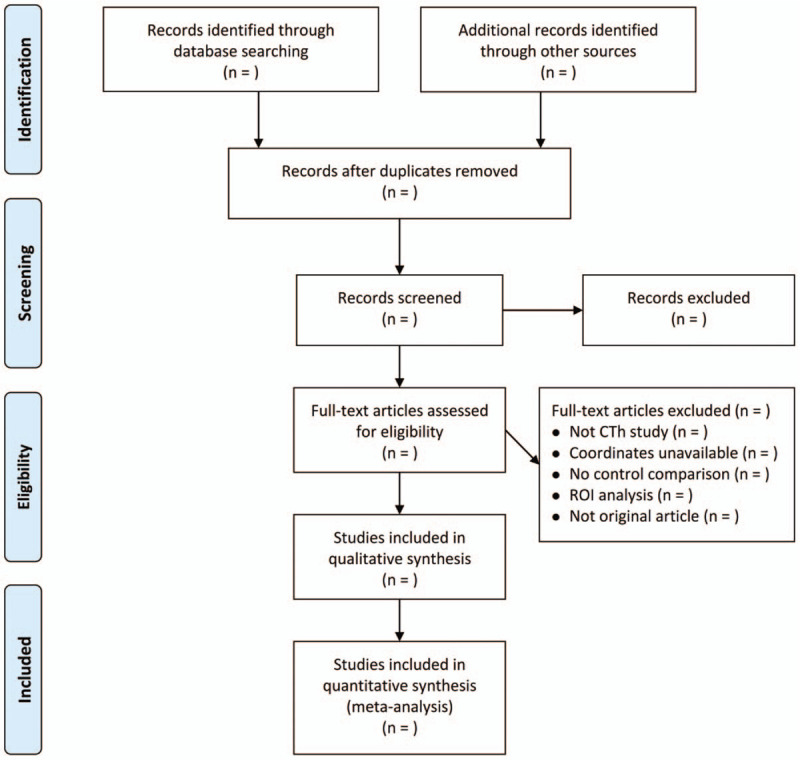
Flowchart of study selection following the PRISMA. CTh = cortical thickness, HC = healthy control, PRISMA = Preferred Reporting Items for Systematic review and Meta-Analysis, ROI = region of interest.

Figure 1 presents the flowchart of study selection following the PRISMA.^[27]^

### 2.3 Data extraction

Data abstracted from the eligible studies will be: the first author's name, year of publication, sample size, age, sex distribution, CP subtype, pain duration, pain intensity, magnetic resonance imaging (MRI) scanner manufacturer and platform, field strength, head coil, MRI sequence, repetition time (TR)/echo time (TE), voxel size, imaging processing software package, smooth kernel, statistical model, covariate, statistical threshold, peak coordinates, height of the peaks (t-values, z-values, or *P*-values), their stereotactic reference space (MNI or Talairach), and quality control.

### 2.4 Quality assessment

Quality assessment of each included study will be performed using a 12-point checklist based on a previous CTh meta-analysis (details in Table 1).^[28]^ The items utilized for the quality assessment were categorized into 3 parts: subjects (4 points), methods for imaging acquisition and analysis (5 points), and results and conclusions (3 points).

**Table 1:**
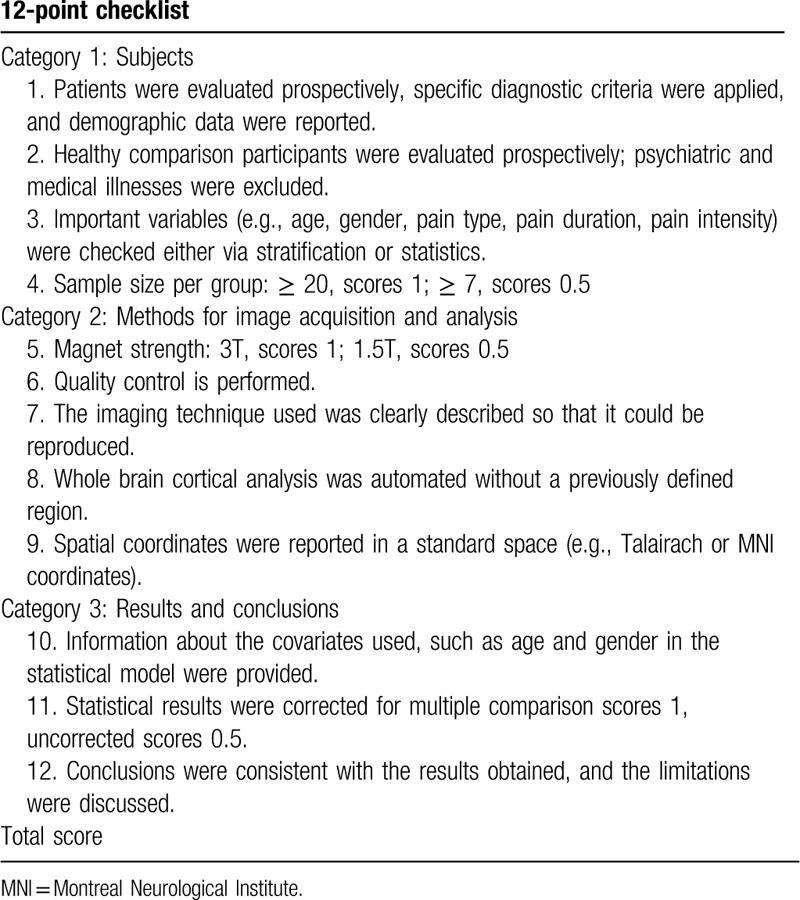
The checklist of quality assessment for the included cortical thickness studies.

Two investigators will independently perform literature search, study selection, data extraction, and quality assessment. Any disagreements will be resolved by a consensus-based discussion.

### 2.5 Main coordinate-based meta-analysis (CBMA)

The SDM-PSI software package (version 6.21, https://www.sdmproject.com/) will be employed to conducted this main CBMA to identify consistent CTh differences between patients with CP and pain-free controls. The detailed processing steps can be found in the SDM-PSI reference manual (https://www.sdmproject.com/manual/) and other publications.^[24,25,29]^ To obtain the meta-analytic results, a correction for multiple comparison: threshold-free cluster enhancement family wise error rate (TFCE FWER) with a *P* < .05 and a minimum cluster size ≥ 10 voxels, will be utilized.^[24,25]^

### 2.6 Reliability analysis

Sensitivity analysis will be conducted to assess the stability of the results identified in the main CBMA.

Heterogeneity analysis of significant results will be performed using the I^2^ statistic.

Publication bias will be examined using the Egger test.^[30]^ A threshold at *P* < .05 will be considered significant.

### 2.7 Subgroup analysis

Subgroup CBMA will be performed in clinical subtypes, different MRI field strengths (3.0T and 1.5T MRI), and different software packages for CTh analysis if the corresponding number of the datasets is sufficient (n ≥ 10).

### 2.8 Meta-regression analysis

Meta-regression analyses will be carried out to examine if regional CTh alterations across studies were confounded by age, gender, pain duration, and pain intensity if they were available from at least 10 datasets. Threshold-free cluster enhancement family wise error rate (TFCE FWER) with a *P* < .05 and a minimum cluster size ≥ 10 voxels will be employed to determine statistical significance.^[24,25]^

### 2.9 Ethics and dissemination

Because we will use data from published studies, no Ethics approval or patient consent is required in this meta-analysis. We will publish the results of this meta-analysis in a peer-reviewed scientific journal.

## 3 Discussion

To the best of our knowledge, this will be the first CBMA of SBM studies that characterizes brain CTh alterations in CP. The reliability and reproducibility of the results from neuroimaging research have been increasingly concerned.^[31]^ Many confounding factors, such as small sample size, variety in sample characters and etiologies, and differences in magnetic resonance imaging (MRI) scanner manufacturer and platform, field strength, imaging data acquisition parameter, imaging processing software package, smooth kernel, statistical model, covariate, and statistical threshold used, may cause the low reliability and reproducibility. Further investigations are necessary to reduce these impacts. This CBMA will provide quantitative evidence of common brain cortical morphometry of CP. The findings will help us to understand the neural basis underlying CP.

## Author contributions

**Conceptualization:** HaiRong Ma, ZhenYu Dai, PingLei Pan

**Data curation:** HaiRong Ma, LiQin Sheng

**Formal analysis:** HaiRong Ma, LiQin Sheng

**Funding acquisition:** PingLei Pan

**Investigation:** HaiRong Ma, LiQin Sheng, Fei Chen

**Methodology:** LiQin Sheng, CongHu Yuan

**Project administration:** Fei Chen

**Resources:** LiQin Sheng, Fei Chen, CongHu Yuan

**Software:** LiQin Sheng

**Supervision:** ZhenYu Dai

**Validation:** Fei Chen, PingLei Pan

**Visualization:** LiQin Sheng, HaiRong Ma

**Writing – original draft:** HaiRong Ma

**Writing – review & editing:** ZhenYu Dai, PingLei Pan
